# Abnormal Presentation of Extrapulmonary Tuberculosis

**DOI:** 10.7759/cureus.31390

**Published:** 2022-11-11

**Authors:** Arju Fatema B Lakhani, Swapnil Date, Sanjay V Deshpande, Prashanth Balusani

**Affiliations:** 1 Department of Orthopaedics and Traumatology, Jawaharlal Nehru Medical College, Datta Meghe Institute of Medical Sciences, Wardha, IND

**Keywords:** newer modalities, extrapulmonary tuberculosis (eptb), biomarker, tuberculosis, tubercular arthritis, wrist arthritis

## Abstract

Tuberculosis is a common bacterial infection that mainly affects the respiratory system; however, it can involve other structures such as lymph nodes, pericardium, pleura, central nervous system, gastrointestinal system, and skeletal system. Skeletal tuberculosis is secondary to pulmonary and abdominal tuberculosis. Skeletal involvement generally involves the vertebral column, hip, and knee joint. Tuberculosis of small peripheral joints is an uncommon entity. In this report, we report tubercular arthritis of the wrist joint in a 40-year-old female patient who presented with swelling and pain in the wrist joint.

## Introduction

Tuberculosis remains one of the concerning health topics for developing as well as developed nations [1]. Earlier, pulmonary tuberculosis (PTB) had attracted the attention of various researchers. However, after the 1980s, more attention was drawn to extrapulmonary manifestations of tuberculosis, involving sites like lymph nodes, pericardium, pleura, bones, and joints along with the central nervous system and gastrointestinal systems. Earlier patients' immunocompromised state was thought to be a reason behind these extrapulmonary involvements. Nevertheless, studies showed that immunocompetent patients could also have extrapulmonary manifestations [2,3], in which the most predominant sites involved are lymph nodes, pleura, gastrointestinal tract, bone, central nervous system (CNS), and genitourinary system [4]. It is documented that about 10% to 15% of total tuberculosis cases are extrapulmonary. Among these one-tenth affect the skeletal system [5]. Reports have also shown data of patients in whom the tests done for tuberculosis (acid-fast stain, lack of granuloma on histopathological examination, culture of the bacteria, along with GeneXpert) were negative; still, that does not rule out the diagnosis of tuberculosis, since these patients have shown improvement by taking anti-tubercular drug regimen [3].

## Case presentation

A 40-year-old female came to the outpatient department of the orthopaedic department of a tertiary care hospital, with chief complaints of pain and swelling around her left wrist for two years. The pain was insidious in onset, on and off type, dull aching in nature, which had recently been progressive for the past six months. The pain was non-radiating, moderate in severity, aggravated by movements and decreased on taking medications and rest, which would progress and regress over two years but did not subside entirely; a history of occasional fever was present, with considerable weight loss. No history of trauma, diurnal variation, or other constitutional symptoms was noted. The patient had been treated in a local hospital, where she was prescribed antibiotics and analgesics. However, the pain was not relieved when the patient came to the tertiary care centre.

Clinical examination findings 

On Clinical examination of the primary site, diffuse swelling, which was boggy in consistency, ill-defined, smooth, present over the dorsum of the wrist, with tenderness over the radiocarpal joint and carpals was found. The swelling was freely mobile, i.e., it was not attached to underlying structures or overlying skin, there was no raised temperature, overlying skin was smooth, no pulsations were seen over the swelling, swelling didn't change size with the position of the upper limb, fluctuation test was positive, transillumination test was negative, size of the swelling was 5 x 3 x 2 cm. Gross muscle wasting of the forearm, thenar and hypothenar muscles was seen. The exact range of wrist and finger movements was not appreciated because of the pain. However, the approximate range of movements wrist was taken into consideration showing dorsiflexion zero to 30 degrees, palmar flexion zero to 20 degrees, circumduction, and adduction movements were restricted because of tenderness. For further investigations, aspirate was sent for culture sensitivity, which was positive for bacteria. Investigations were done, including a complete blood count showing a hematocrit (%) of 100 and an erythrocyte sedimentation rate of 46 mm/hr. Haemoglobin was 7.6%, and the tuberculin test was positive (which aroused the suspicion of tuberculosis, which was further confirmed on histopathological examination), though the sputum examination was negative.

Management

For surgical management, the wrist joint was exposed by a 7 cm straight incision centring over the wrist. It was taken over the dorsal aspect of the wrist over the third extensor compartment. Underlying fascia, soft tissue was dissected and retracted. The extensor retinaculum was incised to reach the wrist and carpal bones. Extensive synovial hypertrophy was seen. All hypertrophied synovium adjacent to wrist and carpal bones was excised and sent for histopathological examination. There was destruction of articular cartilage noted over the distal radius. Also, erosions were noted over the scaphoid, lunate, and trapezium bones. Caseous material was seen along the bony erosions, which was excised and sent for histopathological examination. Thorough debridement and irrigation were done and the incision was then closed in layers. Post-operatively, the patient was given injection ceftriaxone 1 gm iv bd for three days followed by tablet cefuroxime 500 mg bd for five days. For pain control, tablet aceclofenac 200 mg, to be taken if necessary, was prescribed. Histopathological examination of the patient showed chronic granulomatous infection and since there was a history of complaints of two years, raised erythrocyte sedimentation rate (ESR) and radiological evidence of destruction along with the patient’s histopathological examination showing chronic granulomatous lesions suggestive of tuberculosis, so oral anti-tubercular treatment was started as an intensive phase having isoniazid (300mg), pyrazinamide (1500mg), rifampicin (600mg), and ethambutol (800mg) in an intensive and continuous phase manner according to National Tuberculosis Elimination Programme (NTEP) regime. The patient was also prescribed nutritional supplements including iron, folic acid, a high-protein diet, and multivitamins. After the surgical procedure, the joint was supported with braces. For supportive management, post-operative physiotherapy and rest were recommended.

Radiographical findings on plain X-ray

**Figure 1:**
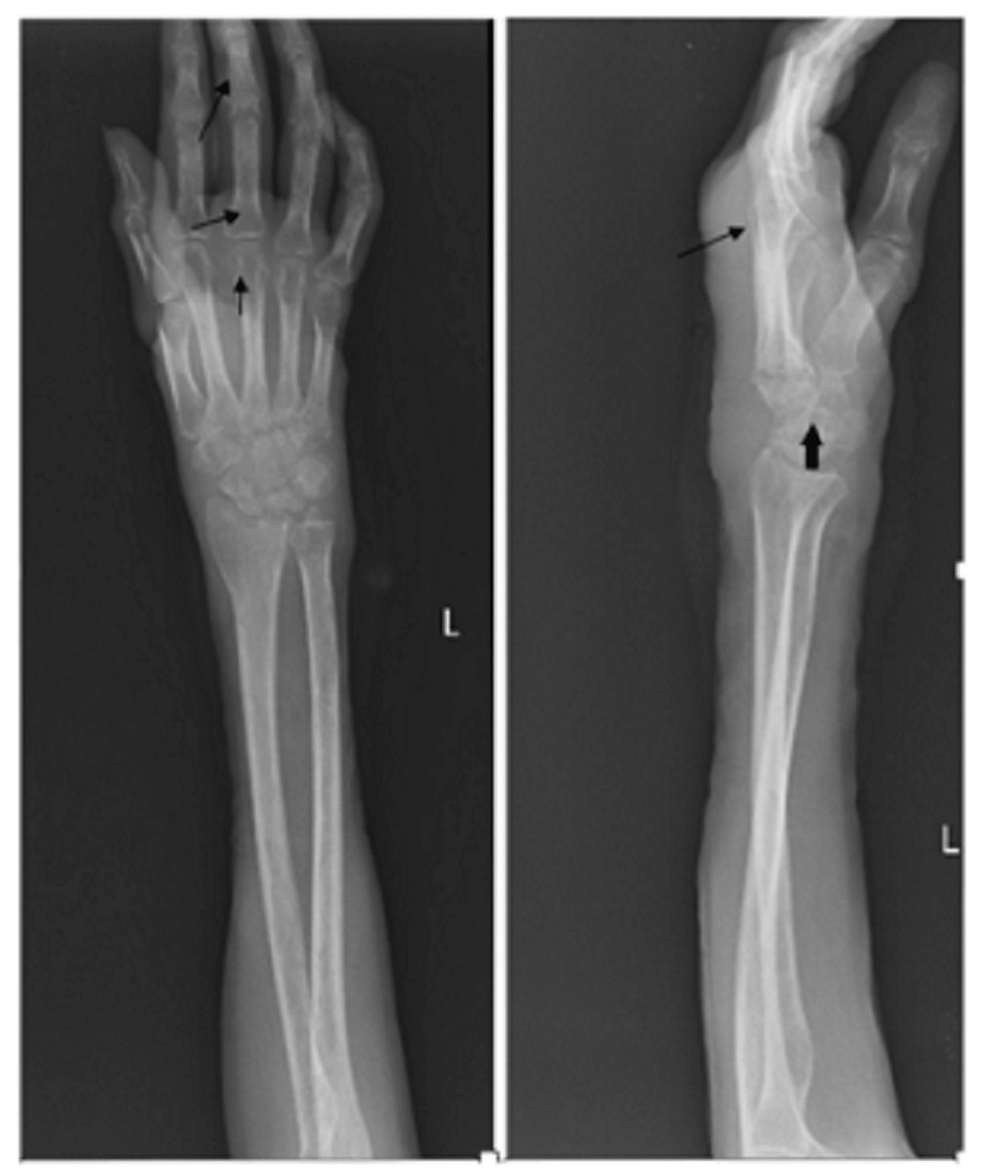
Left side showing anteroposterior (AP) view and right side showing lateral view

There was diffused soft swelling seen in the lateral view and anteroposterior aspect. Joint space was reduced. Articular margins of the distal end of the radius and scaphoid were irregular and destroyed. Carpal bones lost their shape and smooth surfaces were seen. The base of the second, third, and fourth metacarpal was destroyed. Radiocarpal, intercarpal, and carpometacarpal joints were not differentiable (Figure 1). Chest radiogram posteroanterior (PA) view was normal.

Histopathological examination

**Figure 2:**
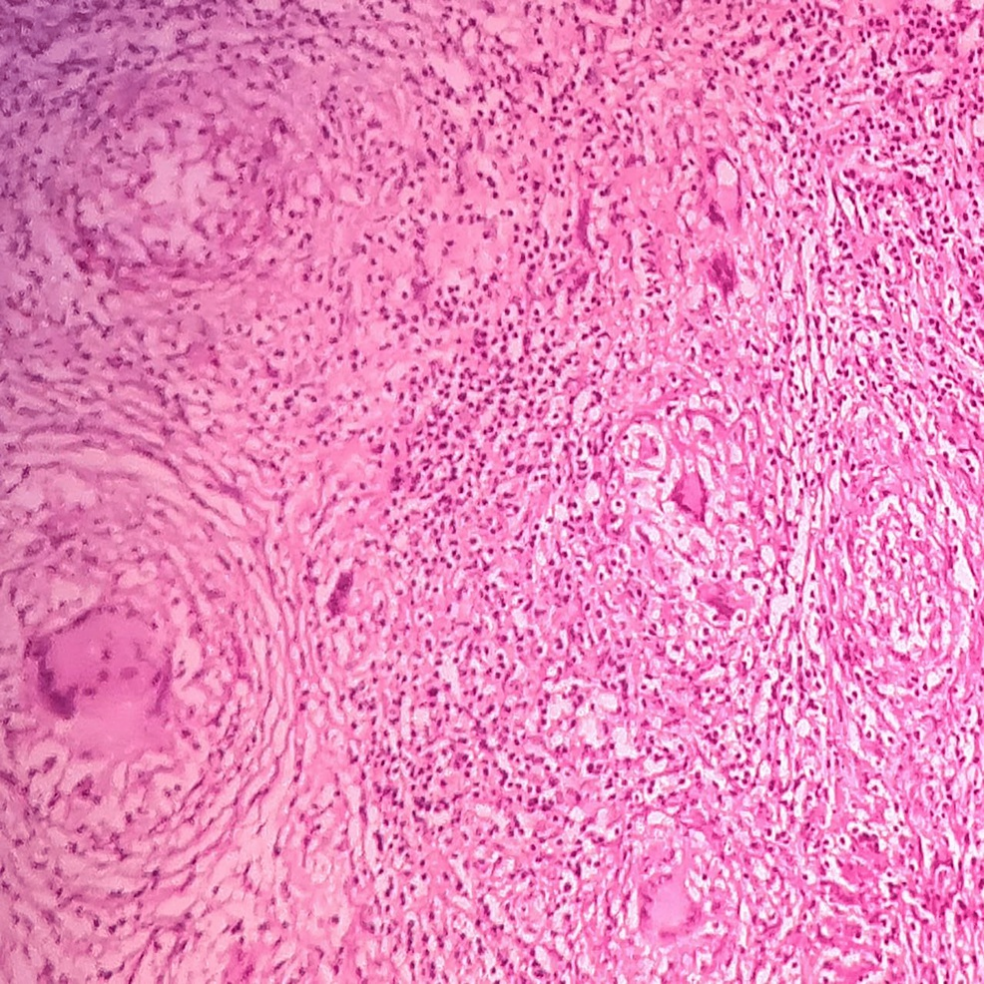
Histopathological examination showing tubercular granulomatous lesion

On histopathological examination, the given section stained with hematoxylin and eosin stain (low power view: 10x) showed the formation of central caseous necrosis surrounded by fibroblasts, epitheloid cells, Langhans giant cells, and lymphocytes. Histopathological features were suggestive of chronic granulomatous lesion tuberculosis (Figure 2).

Current condition

Now the patient is symptomatically better. There is no recurrence of swelling. The patient still experiences dull aching on and off pain while performing wrist movements, but the severity of the pain has reduced. The patient is now able to perform wrist flexion from zero to 70 degrees and extension from zero to 50 degrees. The finger grip has also improved. The surgical site is normal. Muscle wasting has improved.

## Discussion

Tuberculosis is one of the common infections we all encounter, and depending on how the person's immunity responds to the infectious agent, the course of the disease is decided, which generally manifests and eventually results in pulmonary tuberculosis in an immunocompromised patient more than in an immunocompetent patient, either pulmonary or extrapulmonary. Mainly in an extrapulmonary tuberculosis patient, the presenting complaints are generally nonspecific or can be said as non-classic, which prevents us from clenching the diagnosis; so the diagnosis of tuberculosis in a patient is often missed resulting in a delay in the initiation of appropriate treatment. To be specific, suppose there are manifestations of extrapulmonary tuberculosis or tubercular arthritis, in such cases, it is usually misdiagnosed as pain due to any physical trauma, gout, or rheumatoid arthritis [6-8]; this is also because radiological signs are not well established in early stage and gradually evolve with further progression of the disease [9].

The most commonly affected joints are the weight-bearing joints [6], while in our case, we are discussing a case of tubercular arthritis of the wrist joint, which is a non-weight bearing joint. In such instances, culture is considered a gold standard diagnostic modality [10]. Still, there are cases reported showing negative culture reports, which still had tuberculosis [11]; in this report, we have discussed a case in which the patient's sputum was negative for acid-fast bacilli (AFB) along with showing culture and sputum negativity. In such cases, biomarker-based studies can play a role in the early detection of tuberculosis [10]. Biomarker, along with the diagnosis of tuberculosis, has also been proven efficient enough to detect the progression of the infection and the chances of recurrence of the disease. Other modalities such as T-cell function, T-cell responses, serological studies, and studies based on protein and gene expression seem to be in the race of being the choice of test for diagnosis of the same [12].

Tuberculous arthritis is an uncommon condition which needs proper surveillance for its diagnosis, which includes AFB smear, culture and histology and a combined medical and surgical approach [6]. With the development of diagnostic modalities, newer treatment modalities also seem to be developing for the treatment of extrapulmonary tuberculosis right from the introduction of the targeting phenomenon first introduced for the treatment of Tuberculosis by Paul Ehrlich, considered hypothetically as a “magic pill”, which further developed into targeted drug delivery system including active targeting drug delivery system and passive targeting drug delivery [13], to the use of serum CXCR3 ligands as biomarkers for the treatment monitoring of tuberculosis [14]. All of it shows the efforts of researchers. However, the problem remains in tuberculosis-endemic regions where the facilities of diagnostic and treatment remain limited due to cost or demand and supply imbalance and even if there is a diagnosis, is there a patient-specific treatment [15]? Will these new modalities help to change the present scenario of the health system? If yes, when?

## Conclusions

The extrapulmonary presentation of tuberculosis is a type of tuberculosis where instead of the respiratory system, some other site or organs are involved; it can be both primary or secondary in nature. In this report, the centre of the discussion is the skeletal involvement of tuberculosis. In this case report, we are discussing the abnormal presentation of extra-pulmonary tuberculosis showing manifestation at the level of the wrist joint; here we have tried to show the line of management and how the diagnosis is clenched. 
